# The Role of Microbiota-Related Co-Metabolites in MASLD Progression: A Narrative Review

**DOI:** 10.3390/cimb46070381

**Published:** 2024-06-25

**Authors:** Maria Martin-Grau, Daniel Monleón

**Affiliations:** 1 Department of Pathology, University of Valencia, 46010 Valencia, Spain; 2 University Clinical Hospital of Valencia Research Foundation (INCLIVA), 46010 Valencia, Spain

**Keywords:** microbiota, metabolites, MASLD, SCFAs, hippurate, indoles, TMAO, BCAAs, polyamines, BAs

## Abstract

Metabolic dysfunction-associated steatotic liver disease (MASLD) represents a growing health concern due to its increasing prevalence worldwide. Metabolic homeostasis encompasses the stable internal conditions vital for efficient metabolism. This equilibrium extends to the intestinal microbiota, whose metabolic activities profoundly influence overall metabolic balance and organ health. The metabolites derived from the gut microbiota metabolism can be defined as microbiota-related co-metabolites. They serve as mediators between the gut microbiota and the host, influencing various physiological processes. The recent redefinition of the term MASLD has highlighted the metabolic dysfunction that characterize the disease. Metabolic dysfunction encompasses a spectrum of abnormalities, including impaired glucose regulation, dyslipidemia, mitochondrial dysfunction, inflammation, and accumulation of toxic byproducts. In addition, MASLD progression has been linked to dysregulation in the gut microbiota and associated co-metabolites. Short-chain fatty acids (SCFAs), hippurate, indole derivatives, branched-chain amino acids (BCAAs), and bile acids (BAs) are among the key co-metabolites implicated in MASLD progression. In this review, we will unravel the relationship between the microbiota-related metabolites which have been associated with MASLD and that could play an important role for developing effective therapeutic interventions for MASLD and related metabolic disorders.

## 1. Introduction

Metabolic dysfunction-associated steatotic liver disease (MASLD) has emerged as a significant public health concern due to its increasing prevalence worldwide [1]. MASLD is a liver disease characterized by a spectrum of stages, including simple steatosis, hepatic inflammation (known as metabolic dysfunction-associated steatohepatitis (MASH)), fibrosis, cirrhosis, and ultimately hepatocellular carcinoma (HCC). The recent redefinition of the term MASLD from the non-alcoholic fatty liver disease (NAFLD) has highlighted the metabolic dysfunction that characterize the disease, involving multiple factors that influence and contribute to it [2,3]. Among these contributors, the gut microbiota and its associated metabolites have become important players in the development and progression of this liver disease.

The gut microbiota plays a crucial role in maintaining host health. It is composed of trillions of microorganisms, where bacteria, virus, fungi, and parasites share a common habitat [4]. They are subjected to the dietary nutrients they are going to have access to, allowing them to grow, divide, interact among other microorganisms, and finish the digestion of several compound that eukaryote cells will absorb. The metabolites derived from the gut microbiota metabolism can be defined as microbiota-related co-metabolites. They serve as mediators between the gut microbiota and the host, influencing various physiological processes. The microbiota-related co-metabolites can be characterized using metabolomics and can be measured via nuclear magnetic resonance (NMR) or mass spectroscopy (MS) [5]. This omics discipline focuses on studying the metabolism of a biological sample, where the metabolites resulting from metabolic processes are determined. MASLD has been extensively studied using animal models. It has been observed that the consumption of high-calorie diets such as high-fat diets (HFD), high-cholesterol high-fat diets, or high-fructose diets promotes the development of various stages of MASLD as well as the onset of different comorbidities, such as insulin resistance (IR), obesity, and type 2 diabetes mellitus (T2DM) [6]. Additionally, the consumption of these diets has also been associated with changes in the gut microbiota [7]. In this review, we will unravel the relationship between the main microbiota-related co-metabolites detected by metabolomics which have been described in MASLD studies induced by genetic, chemical, or dietary interventions. Microbiota-related co-metabolites can be classified depending on their chemical nature and origin. In the following paragraphs, microbiota-related co-metabolites derived from carbohydrate metabolism (methanol, formate, ethanol, lactate, short-chain fatty acids (SCFAs), malonate), those derived from vitamin metabolism (choline- trimethylamine *N*-oxide (TMAO) axis), those derived from aromatic compound metabolism (hippurate), those exclusively derived from protein metabolism (homovanillate, *N*-phenylacetylglycine, indole-*3*-acetic acid (IAA), branched-chain amino acids (BCAAs), polyamines), and those related to bile acid (BA) metabolism will be described. Their study and understanding could play an important role in proposing new biomarkers for the detection of MASLD.

## 2. Microbiota-Related Co-Metabolites Derived from Carbohydrate Metabolism

**Figure 1:**
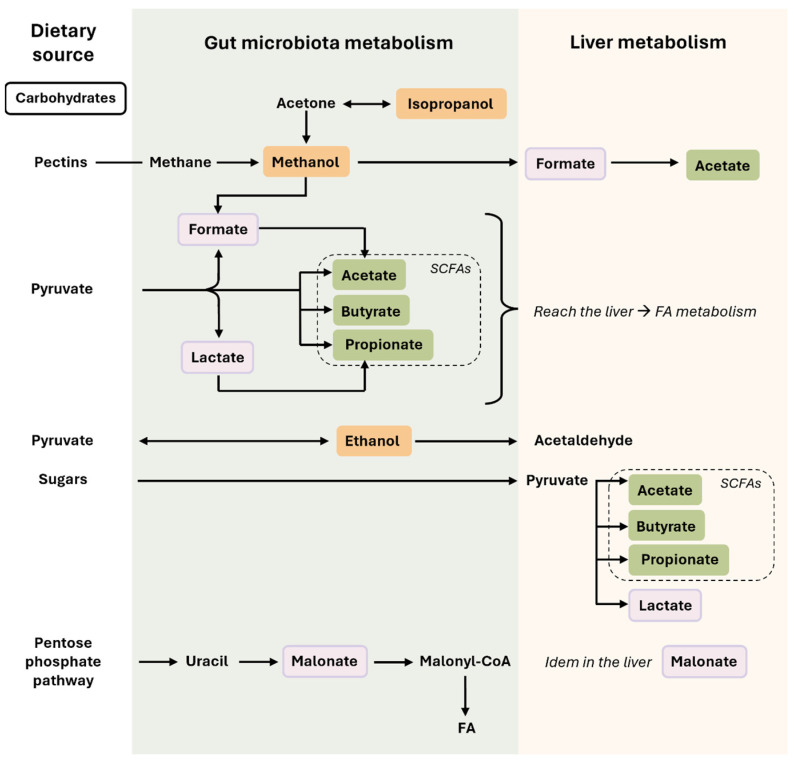
Microbiota-related co-metabolites derived from carbohydrate metabolism. In this figure, we can observe how nutrients derived from carbohydrates are transformed in the intestine (by the action of the gut microbiota) or in the liver. Some compounds, such as SCFAs, can be produced by both the gut microbiota and hepatocytes. The colors used for the microbiota-related co-metabolites highlight their different nature and origin, which are described in the main text. Briefly, rectangles in orange are the alcohols compounds (methanol, isopropanol, and ethanol), in green the SCFAs, and in light purple the acids derived from carbohydrate metabolism (lactate, formate, malonate). Single-headed arrows indicate irreversible steps, while double-headed arrows indicate that the process can be reversible. Abbreviations: FAs, fatty acids; SCFAs, short-chain fatty acids.

Carbohydrates that escape digestion by host enzymes in the stomach are utilized by microorganisms, producing an array of metabolites such as alcoholic compounds, derivatives of pyruvate fermentation, or derivatives of the pentose phosphate pathway (Figure 1).

### 2.1. Alcohol Compounds: Methanol–Formate Axis

Alcohols are compounds that contain a hydroxyl (-OH) group in their structure. Eukaryotic cells cannot synthesize them, and their presence may result from direct absorption (exogenous source) or from endogenous production by microorganism fermentation. The liver, an organ containing the necessary enzymes for their elimination, can metabolize them.

Methanol, a byproduct of methane metabolism, is synthesized from the fermentation of pectins [8]. Its production is carried out by methanogenic microorganisms such as *Bacteroides* and *Methanobrevibacter* [8,9]. Methanol can be transformed into formate, which can be directly utilized for acetate synthesis. Moreover, formate is involved in one-carbon metabolism, which is necessary for the synthesis of nucleotides and amino acids [10]. The accumulation of methanol can be toxic for eukaryote cells, but its metabolic conversion to other intermediates is crucial for eliminating it [11]. Methanol and formate were detected in the liver metabolic profile of leptin-deficient obese (*ob*/*ob*) mice that presented steatosis [12].

Furthermore, isopropanol is also an alcohol compound that bacteria can produce and convert to methanol via conversion into acetone, which can participate in SCFA synthesis. Microorganisms such as *Clostridium* are related to isopropanol synthesis [13]. Isopropanol has been described in the urine of mice fed a HFD for 12 weeks [14].

### 2.2. Pyruvate Fermentation Derivatives: Ethanol, Lactate, and SCFAs

Pyruvate is the product of glycolysis. Bacteria ferment pyruvate to generate ethanol, lactate, or SCFAs in the absence of oxygen.

Ethanol is also an alcohol compound, whose endogenous production by microorganisms is greatly influenced by a diet’s composition [15]. Microorganisms such as *Ruminococcus*, *Weisella*, or *Klebsiella* are associated with its synthesis [9,15,16,17]. Ethanol can reach the liver, where it will be metabolized by liver enzymes, enhancing the host–microbiota interaction. Ethanol degradation will produce an intermediate compound that is toxic for eukaryote cells—acetaldehyde. Changes in ethanol levels produced by microorganisms have been described in the spectrum of MASLD stages, contributing to liver toxicity [9,16,18].

Lactate is generated through the action of a dehydrogenase, a reaction that occurs in microorganisms or eukaryotic cells [19]. Certain microorganisms like lactobacilli, streptococci, and bifidobacteria are known to produce lactate in the gut, while other groups can metabolize it into propionate [20]. Lactate serves as a significant indicator of metabolic activity and displays essential physiological effects in the cells and microorganisms [19,21]. Due to its acidic nature, lactate can contribute to modulate the pH in the gut, lowering it, and it is involved in the absorption of various compounds [21]. Indeed, differences in the acid/base balance can significantly impact the diversity of a bacterial microbiota [20,22]. In MASLD, a decrease in the *Lactobacillus* and *Bifidobacterium* genera has been observed [16,23,24]. Moreover, a reduction in lactate levels has been noted in the feces of rats fed a HFD for 12 weeks [25], suggesting potential alterations in the diversity of lactate-producing and lactate-consuming microbes, as well as changes in intestinal pH values.

SCFAs are fatty acids with a short carbon chain, primarily consisting of acetate (2 carbons), propionate (3 carbons), and butyrate (4 carbons). All of them can be synthesized from pyruvate, and acetate can be synthesized from formate and propionate from lactate. Their synthesis has been described in the colon through the fermentation of carbohydrates by the gut microbiota [16,26,27]. Among the bacteria involved, we find genera such as *Akkermansia*, *Bifidobacterium*, *Blautia*, *Coprococus*, *Eubacterium*, *Lactobacillus*, *Prevotella*, *Ruminococcus*, and *Streptococcus* [9,28,29,30]. SCFAs have been shown to exert anti-inflammatory and beneficial metabolic effects. First, they increase mucus secretion in the intestine, and they decrease luminal pH due to their acidic nature. Secondly, SCFAs can be used by enterocytes to obtain energy through beta-oxidation, and they can be used by the endocrine intestinal L-cells to synthesize gut hormones such as the glucagon-like peptide-1 (GLP-1). GLP-1 actions influence different tissues, such as white adipose tissue (WAT), or the liver, promoting a decrease in blood glucose levels, increasing the energy expenditure, stimulating insulin secretion, and decreasing the release of pro-inflammatory cytokines [31]. Moreover, the presence of SCFAs has been associated with microbiota related to metabolic health [22,31]. It has been described that diets based on HFD and ones that are low in fiber can modulate gut microbiota diversity, changing it to an aberrant microbiota unable to synthesize SCFAs and promoting a pro-inflammatory environment [31]. In fact, the supplementation with SCFAs decreased HFD-induced steatosis in mice [32]. Furthermore, it alleviated the pro-inflammatory environment and decreased serum transaminase levels in a methionine-choline-deficient diet in a MASH mice model [33].

### 2.3. Pentose Phosphate Pathway Derivatives: Malonate

The pentose phosphate pathway is done by bacteria or eukaryote cells to generate sugars necessary for DNA synthesis or intermediates of metabolic pathways [29]. Malonate is a small organic compound linked to pyrimidine metabolism, specifically in the breakdown of uracil by bacteria [34]. Additionally, it plays a role in fatty acid (FA) synthesis, serving as a precursor for malonyl-CoA synthesis, a crucial component in FA elongation and lipid metabolism. Moreover, it acts as a competitive inhibitor of enzymes in the tricarboxylic acid (TCA) cycle, particularly succinate dehydrogenase. Inhibiting this enzyme can disrupt the normal flow of electrons and energy production in the mitochondria [35]. There was little information about the role of malonate in the gut–liver axis and MASLD progression. However, an increase in malonate was observed in feces from a HFD-induced steatosis rat model [25], suggesting an alteration in the pentose phosphate pathway derivatives and microbiota metabolism. Moreover, a significant increase in uridine, which can be transformed into uracil, was described in an (*ob*/*ob*) steatosis mice model [12].

## 3. Microbiota-Related Co-Metabolites Derived from Vitamin Metabolism

### Choline–TMAO Axis

**Figure 2:**
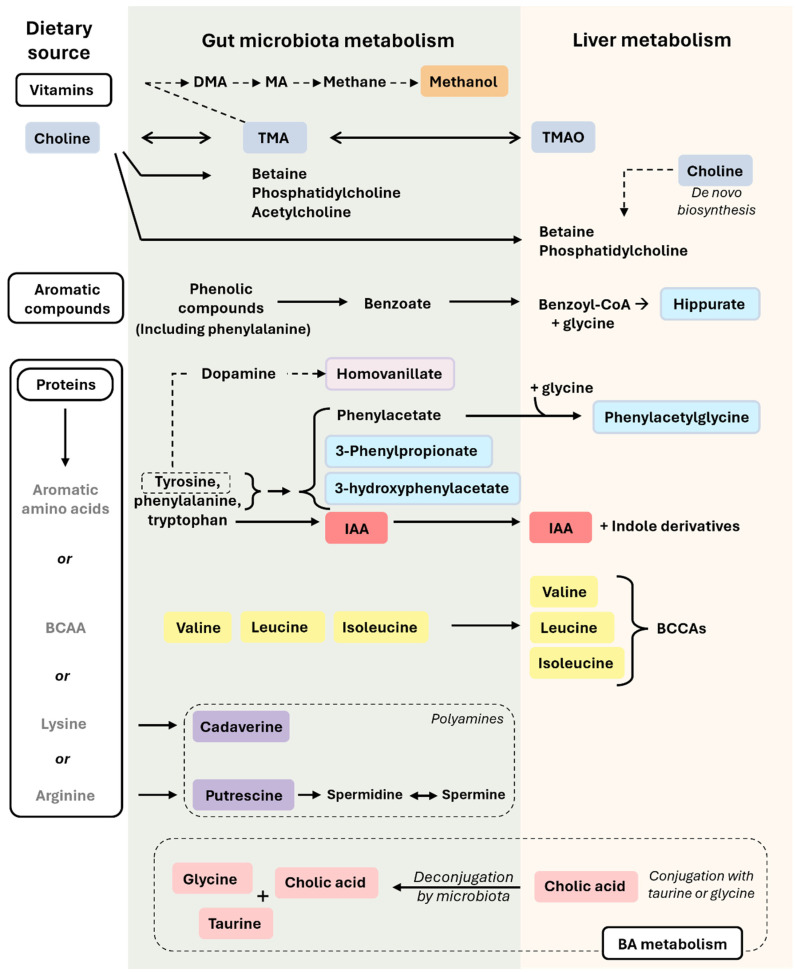
Microbiota-related co-metabolites derived from the metabolism of vitamins, aromatic compounds, protein, and BA. In this figure, we can observe how nutrients derived from vitamins, aromatic compounds, proteins, and BA are transformed in the intestine (by the action of the gut microbiota) or in the liver. Metabolites from the gut will reach then the liver. The colors used for the microbiota-related co-metabolites highlight their different nature and origin, which are described in the main text. Briefly, rectangles in orange refer to methanol (also seen in Figure 1), in dark blue to choline metabolism (choline, TMA, TMAO), in light blue to compounds derived from tyrosine, phenylalanine, and other aromatic compound metabolism (hippurate, *N*-Phenylacetylglycine, 3-Phenylpropionate, 3-hydroxyphenylacetate), in light purple to metabolites derived exclusively from tyrosine metabolism (homovanillate), in red to metabolites exclusively from tryptophan metabolism (IAA), in yellow to BCAAs (valine, leucine, isoleucine), in purple to polyamines (cadaverine, putrescine), and in pink to BA metabolism (cholic acid, glycine, taurine). Single-headed solid arrows indicate irreversible steps, while double-headed solid arrows indicate that the process can be reversible. Dashed line arrows refer to only from tyrosine metabolism, homovanillate is produced. Abbreviations: BA, bile acid; BCAAs, branched-chain amino acids; DMA, dimethylamine; IAA, indole-3-acetic acid; MA, methylamine; TMA, trimethylamine; TMAO, trimethylamine-N-oxide. The colors used for the microbiota-related co-metabolites highlight their different nature and origin, which are described in the main text.

Choline is a vitamin that can be used in the gut to synthesize trimethylamine (TMA), phosphatidylcholine, betaine, or acetylcholine via microorganisms. Furthermore, choline can also reach the liver or be synthesized de novo and be used mainly for the synthesis of phosphatidylcholine and betaine [36,37]. In the liver, TMA can be oxidized into TMAO (Figure 2). Focusing on the choline–TMAO axis, the composition of the gut microbiota determines the production of TMA. Dysbiosis in the gut microbiota can result in either increased or decreased TMA production, leading to fluctuations in TMAO levels [38]. TMAO has been related to cardiovascular and metabolic diseases [36,39,40]. Elevated TMAO levels have been linked to MASLD [41]. Interestingly, the supplementation of TMAO has shown to attenuate the progression of liver fibrosis in a mouse model of MASH [42]. Moreover, a significant decrease in TMAO was observed in an (*ob*/*ob*) mice model with steatosis [12]. Even if the role of TMAO seems contradictory, its metabolism is altered in MASLD.

## 4. Microbiota-Related Co-Metabolites Derived from Aromatic Compound Metabolism

Aromatic compounds (including phenolic compounds) and flavonoids are abundant in a variety of plant-based foods, such as fruits, vegetables, herbs, and spices. These compounds offer potential health benefits due to their antioxidant and anti-inflammatory properties [43].

### Hippurate

Hippurate is a metabolite that derives from the phenolic aromatic compound metabolism carried out by the gut microbiota. Its biosynthesis is related to the *Clostridium*, *Faecalibacterium*, *Bifidobacterium*, *Subdoligranulum*, and *Lactobacillus* genera [23,44,45]. Initially, benzoate, derived directly from the breakdown of dietary aromatic compounds by bacteria, is absorbed. Subsequently, in the liver, benzoate undergoes a transformation into benzoyl-CoA, which is then conjugated with a glycine to produce hippurate (Figure 2). Finally, hippurate is transported to the kidneys for excretion via urine [44]. Due to its conjugation with an amino acid and its presence in urine, hippurate plays a crucial role in nitrogen surplus elimination and host–microbiota co-metabolism. In fact, high levels of hippurate were proposed as a marker of gut microbiome diversity [46] and as a general marker of metabolic health [47]. A significant reduction in hippurate levels was observed in a general MASLD model [48] and fibrosis model [49], suggesting a decrease in gut microbiome diversity and metabolic health in the animals that were treated.

## 5. Microbiota-Related Co-Metabolites Derived Exclusively from Protein Metabolism

The digestion of proteins generates small peptides or amino acids that will be absorbed by enterocytes or utilized by intestinal bacteria (Figure 2). In the following paragraphs, we will discuss the metabolism of certain amino acids by bacteria and the resulting metabolites.

### 5.1. Aromatic Amino Acid Derivatives

Homovanillate, also referred to as 3-methoxy-4-hydroxyphenyl-acetate, belongs to a class of organic compounds known as methoxyphenols. It is involved in the metabolism of tyrosine and dopamine as an intermediate in the dopamine degradation pathway. Dopamine, a neurotransmitter primarily synthesized in the brain, can also be produced in the intestine by various bacteria, thus influencing the gut–brain axis [29,37,50]. Homovanillate has been associated with several genera, including *Clostridium*, *Faecalibacterium*, *Bifidobacterium*, *Subdoligranulum*, and *Lactobacillus* [23]. Dysbiosis in the gut microbiota can impact cognitive function in various neurological disorders [37]. Furthermore, MASLD patients may experience cognitive impairment due to low dopamine levels, exacerbated by HFD consumption, which decreased dopamine levels in the hippocampus [51]. Additionally, dopamine levels have been linked to SCFAs, with higher SCFA levels enhancing dopamine absorption. Reductions in SCFAs result in dopamine dysregulation, as evidenced by the decreased levels of homovanillate [52].

*N*-Phenylacetylglycine is also involved in the metabolism of dietary aromatic compounds [44,53]. Gut microorganisms metabolize phenylalanine to produce phenylacetate [53]. Subsequently, phenylacetate is converted into phenylacetyl-CoA and then into *N*-Phenylacetylglycine. The conjugation with glycine also occurs in the liver [29]. This compound can later be excreted in urine [49,54]. *N*-Phenylacetylglycine has also been associated with different genera, including *Clostridium*, *Faecalibacterium*, *Bifidobacterium*, *Subdoligranulum*, and *Lactobacillus* [23]. The alteration of the composition of the gut microbiota by antibiotics has been shown to affect *N*-Phenylacetylglycine levels in rat urine [45]. Additionally, in rats with carbon tetrachloride (CCl_4_)-induced chronic hepatotoxicity, there was a significant reduction in *N*-Phenylacetylglycine levels in urine [49].

Indole-3-acetic acid (IAA) is produced by the gut microbiota from dietary tryptophan. Once synthesized in the gastrointestinal tract, IAA can be absorbed into the bloodstream and then undergo hepatic metabolism, leading to the formation of various indole derivatives [55]. Furthermore, the concentration of IAA is directly influenced by the composition and activity of the gut microbiota. This compound has been associated with genera such as *Citrobacter, Clostridium*, *Escherichia*, and *Lactobacillus* [29,55]. In a mouse model of HFD-induced MASLD, the levels of IAA were found to be decreased in the liver [55]. Additionally, indole derivatives have been linked to anti-inflammatory effects. Supplementation with IAA reduces the pro-inflammatory effects of a Western diet by decreasing hepatic steatosis and overall inflammation parameters in mice [56]. Moreover, the supplementation with indole-*3*-propionic acid (IPA), one of the indoles derivatives, inhibited microbial dysbiosis, maintained the intestinal epithelium homeostasis, and reduced the production of pro-inflammatory cytokines and the development of liver steatosis in rats fed a HFD for 8 weeks [57].

In addition, compounds such as 3-phenylpropionate or 3-hydroxyphenylacetate have also been associated with the metabolism of aromatic amino acids and microbiota [23]. For example, an increase in 3-phenylpropionate has already been described in the feces of rats fed a HFD for 12 weeks [25].

### 5.2. Branched-Chain Amino Acids (BCAAs)

Valine, leucine, and isoleucine are known as BCAAs. They are essential for protein synthesis. Furthermore, they can be transformed in the liver into branched chain ketoacids (BCKAs) (Figure 2), which can subsequently enter into the TCA cycle. They are necessary for the energy balance of hepatocytes and other signaling processes in the cell [58,59]. Elevated levels of BCAAs have been linked to conditions such as MASLD, T2DM, and IR [59,60]. Furthermore, BCAAs have been proposed as diagnostic biomarkers in MASLD patients. It has been observed that patients with simple steatosis typically do not show significant alterations in BCAA levels in their blood, whereas those with MASH do [61]. Additionally, it has been noted that plasma BCAA levels vary depending on sex. In the progression of MASLD, there is a decrease in plasma BCAA levels in males, whereas females tend to experience an increase [62]. Additionally, dysbiosis of the microbiota induced by a HFD has been linked to an increased absorption and circulation of BCAAs [60].

### 5.3. Polyamines

Polyamines are organic compounds characterized by the presence of multiple amino groups (-NH_2_) separated by carbon chains. Common polyamines include putrescine, spermine, spermidine, and cadaverine (Figure 2). They can be synthesized by the *Clostridium*, *Campylobacter*, *Peptostreptococcus*, and *Peptococcus* genera [9,23]. Polyamines are primarily known for their unpleasant odor. Nonetheless, they also present important anti-inflammatory effects [63].

Putrescine is a small organic molecule composed of two amino groups separated by a four-carbon chain. It is formed through the decarboxylation of arginine. It serves as a precursor for the synthesis of other polyamines, such as spermidine and spermine, which are crucial for maintaining cell integrity and viability [64]. Moreover, they can modulate intestinal macrophage differentiation, protecting the intestinal epithelial cells [63]. Putrescine levels were significantly decreased in the livers of CCl_4_-treated Wistar rats compared to a control group [65].

Cadaverine is a small organic molecule composed of two amino groups separated by a five-carbon chain. It is produced by the decarboxylation of the amino acid lysine. Cadaverine exhibits a protective effect on the intestinal mucosa against enterotoxins released by specific bacteria [66]. Additionally, an elevation in the excretion of cadaverine in rat feces has been observed in those fed a HFD for 12 weeks [25].

## 6. Host–Microbiota Bile Acid (BA) Co-Metabolism from FA and Amino Acid Metabolism

Beyond their conventional role in lipid digestion [67], BA metabolism is linked to gut health and the cholestatic function of the liver. BA profiling has been proposed as a non-invasive metabolic test to classified MASH patients [61]. Apart from BA profiling, there are interesting metabolites associated with BA metabolism and BA homeostasis, such as glycine, taurine, or deconjugated BA (Figure 2).

Glycine plays a crucial role in amino acid metabolism and is involved in purine, serine, and glutathione metabolism. It is closely associated with BA conjugation [61]. In a rat model with high-fat, high-sucrose, diet-induced hepatic steatosis, supplementation of glycine has been shown to improve the condition [67]. Additionally, alterations in the gut microbiota have been linked to the increased excretion of glycine in rat urine [45].

Taurine is an amino acid that conjugates with BA to form conjugated BA. An increased excretion of taurine was also described in rat urine after the alteration of the gut microbiota [45]. In addition, in a CCl_4_-induced chronic hepatotoxicity model in rats, taurine was significantly increased in the urine of the ones that were treated [49]. Supplementation of taurine in the drinking water of female farnesoid X receptor (*Fxr*)-null mice models decreased transaminase, alkaline phosphatase, and triglyceride levels in serum. It also reduced the presence of histological steatosis and decreased the expression of genes related to FA synthesis and oxidative stress, which are traits of MASLD progression [68].

Cholate, a primary BA, plays a pivotal role in BA metabolism and digestion. Originating in the liver from cholesterol, it undergoes conjugation with an amino acid, typically glycine or taurine, to form bile salts. These salts are essential for emulsifying dietary fats and facilitating their absorption in the small intestine. Within the intestine, bacteria facilitate the deconjugation of bile salts into their components, which can then be re-absorbed and transported back to the liver (enterohepatic circulation) or excreted in feces [69]. Genera such as *Lactobacillus*, *Bifidobacteria*, *Enterobacter*, *Bacteroides*, and *Clostridium* are associated with BA metabolism [23]. MASLD patients exhibit elevated serum levels of BA, along with an increase in secondary BA and a higher abundance of BA-metabolizing gut bacteria [24]. However, increased excretion of cholate has been observed in MASLD [61].

## 7. Updated of the Metabolic Homeostasis and Dysfunction Terms

**Figure 3:**
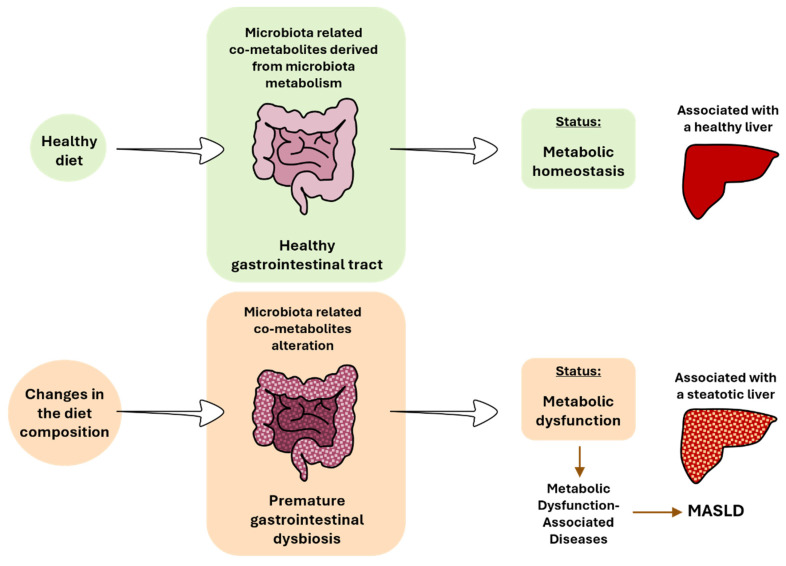
The impact of diet on gut microbiota, gastrointestinal tract, and liver health. MASLD, metabolic dysfunction-associated steatotic liver disease.

The consumption of a healthy diet that is low in saturated fatty acids and high in fiber is associated with proper gastrointestinal function and the optimal functioning of the intestinal microbiota [31]. In this context, the body is in a state of metabolic homeostasis. This concept refers to the maintenance of stable internal conditions related to metabolism within an organism. This involves a balance in various metabolic processes, such as energy production, nutrient utilization, hormone regulation, and waste elimination, to support overall health. Nonetheless, the concept of metabolic homeostasis should comprise a balance in the intestinal microbiota. Achieving equilibrium among different organs also relies on maintaining a balance in bacterial metabolism, considering its impact on other organs (Figure 3).

The change to hypercaloric diets based on high-fat, high-sugar, or high-fat and high-sugar have been associated with premature gastrointestinal dysbiosis. Bacteria will adapt quickly to survive to new dietary sources. Several species will benefit from this new availability of nutrients, while others will not, promoting a change in the microbiota diversity and subsequent derived metabolism pathways. An alteration of the gut microbiota has been described in the different stages of MASLD in rodents and patients [31,32,70,71]. In addition to the changes in the bacterial composition, changes in the mycobiome are beginning to be associated with MASLD [72]. This disruption results in the absence or alteration of key metabolites, contributing to MASLD [16] (Figure 3). The metabolic dysfunction concept arises from an imbalance in metabolic processes, leading to impaired glucose regulation (e.g., insulin resistance), abnormal lipid metabolism (e.g., dyslipidemia), dysfunctional energy production (e.g., mitochondria dysfunction), inflammation (e.g., oxidative stress), and accumulation of toxic byproducts (e.g., reactive oxygen species (ROSs), and oxidized lipids). Generally, metabolic dysfunction is often associated with the development of metabolic disorders, such as T2DM, obesity, and MASLD. In all these metabolic disorders, changes in the gut microbiota have been described, as previously cited. Again, an imbalance in bacterial metabolism should also be considered within this framework.

All the studies mentioned previously have elucidated various metabolic pathways and molecular mechanisms underlying MASLD progression, highlighting the pivotal roles of factors such as the composition of the gut microbiota and microbiota-related co-metabolism. Proportionally, most of the metabolites described here have been found in urine and feces, but they can also appear in blood. Since these are metabolites related to the gut microbiota, it seems logical that they would be altered in feces. However, their presence in urine implies that these metabolites have reached the liver, have been metabolized, and can reach any organ, exerting a local or systemic effect (as reflected in Figure 1 and Figure 2). Urine and feces are easy samples to obtain and present an abundance of possibilities to target specific microbiota co-metabolites for diagnosing and monitoring MASLD. Moreover, recognizing the role of microbiota-related co-metabolites in MASLD opens new avenues for novel therapeutic strategies. Modulating the composition of the gut microbiota through fecal microbiota transplantation, probiotics, prebiotics, phages, or dietary interventions holds promise in preventing and managing MASLD [24,72].

## 8. Conclusions

As research progresses, the intricate relationship between microbiota-related co-metabolites and MASLD becomes increasingly evident. With this review, we have expanded our knowledge on the metabolites related to MASLD and on the metabolic pathways they are involved in. As we have observed, the metabolism processes of carbohydrates, vitamins, proteins, and BAs appear to be the most affected during the development and progression of the disease, impacting the diversity of the gut microbiota and hepatic metabolism. Advancements in the discovery of biomarkers have also provided valuable tools for the early detection and monitoring of MASLD-related pathologies, including hepatic steatosis, inflammation, fibrosis, cirrhosis, and hepatocellular carcinoma. However, there is still no consensus on the use of microbiota-related co-metabolites as a new diagnostic tool in biological samples. Further studies are needed to identify changes in the bacterial diversity within MASLD cohort populations or animal studies and to establish correlations between these bacteria and microbiota-related co-metabolites in the context of MASLD.

## Data Availability

No new data was created. The data presented in this article have been verified using HMDB (https://hmdb.ca/), KEGG pathway (https://www.genome.jp/kegg/), PubChem (https://pubchem.ncbi.nlm.nih.gov/), and PubMed (https://pubmed.ncbi.nlm.nih.gov/), accessed on 17 June 2024.
